# Packed Bed Bioreactor for the Isolation and Expansion of Placental-Derived Mesenchymal Stromal Cells

**DOI:** 10.1371/journal.pone.0144941

**Published:** 2015-12-14

**Authors:** Michael J. Osiecki, Thomas D. Michl, Betul Kul Babur, Mahboubeh Kabiri, Kerry Atkinson, William B. Lott, Hans J. Griesser, Michael R. Doran

**Affiliations:** 1 Institute of Health and Biomedical Innovation, Queensland University of Technology at the Translational Research Institute, Brisbane, Queensland, Australia; 2 School of Chemistry, Physics and Mechanical Engineering, Science and Engineering Faculty, Queensland University of Technology Brisbane, Queensland, Australia; 3 Ian Wark Research Institute, University of South Australia. Adelaide, South Australia, Australia; 4 Mawson Institute, University of South Australia. Adelaide, South Australia, Australia; 5 Department of Biotechnology, College of Science, University of Tehran, Tehran, Iran; 6 University of Queensland Centre for Clinical Research, University of Queensland, Brisbane, Queensland, Australia; 7 Mater Medical Research Institute, University of Queensland, Brisbane, Queensland, Australia; Instituto Butantan, BRAZIL

## Abstract

Large numbers of Mesenchymal stem/stromal cells (MSCs) are required for clinical relevant doses to treat a number of diseases. To economically manufacture these MSCs, an automated bioreactor system will be required. Herein we describe the development of a scalable closed-system, packed bed bioreactor suitable for large-scale MSCs expansion. The packed bed was formed from fused polystyrene pellets that were air plasma treated to endow them with a surface chemistry similar to traditional tissue culture plastic. The packed bed was encased within a gas permeable shell to decouple the medium nutrient supply and gas exchange. This enabled a significant reduction in medium flow rates, thus reducing shear and even facilitating single pass medium exchange. The system was optimised in a small-scale bioreactor format (160 cm^2^) with murine-derived green fluorescent protein-expressing MSCs, and then scaled-up to a 2800 cm^2^ format. We demonstrated that placental derived MSCs could be isolated directly within the bioreactor and subsequently expanded. Our results demonstrate that the closed system large-scale packed bed bioreactor is an effective and scalable tool for large-scale isolation and expansion of MSCs.

## Introduction

Mesenchymal stem/stromal cells (MSCs)-based therapies have potential utility in the treatment of inflammatory diseases, the direct regeneration of mesenchymal tissues, or the up-regulation of innate tissue repair processes [1]. The most widely studied and best characterized MSCs are derived from bone marrow [2]. However, MSCs can be isolated from other tissues that may be more accessible, including placenta, adipose tissue and umbilical cord [3–5]. Placental-derived MSCs (pMSCs) are an attractive source of MSCs, as they not only behave similarly to bone marrow derived MSCs [5], but a single placenta (500–700 g tissue) is sufficient for manufacturing several hundred units of allogeneic MSCs [6].

Regardless of the tissue source, MSC populations will require *in vitro* expansion to generate clinically relevant cell numbers. Many promising therapies require single or multiple doses of approximately 2 x 10^6^ cells/kg [7]. For MSC-based therapies to become a routine and economically viable treatment approach, the most efficient and cost effective method for their large-scale manufacture will require an automated closed-system bioreactor.

Bioreactor designs used for MSC expansion include micro-carrier suspensions in spinner flasks, stirred tank reactors, and perfusion reactors, such as fixed beds or hollow fibre bioreactors [6,8–10]. Simple micro-carrier suspension cultures achieve a large surface area for adherent cell culture. However, there is no connectivity between individual micro-carriers, and empty micro-carriers do not contribute to the total surface area available to the culture. As a result, some micro-carriers rapidly reach confluence, whilst others remain empty; this requires frequent passaging to overcome localized space limitations [6,11]. Micro-carrier cultures also require mixing to enable nutrient exchange and prevent concentration gradients. The shear forces arising from mixing must be carefully modulated, as this can compromise MSC stemness characteristics during expansion [12,13]. Packed bed bioreactors potentially solve both problems by providing a continuous and connected surface area with no need for mixing. However, the maximum perfusion flow velocity cannot exceed 3 x 10^−4^ m/s without compromising the growth rate [14]. This greatly limits the scalability, as both soluble nutrients and oxygen must be supplied by medium perfusion alone.

The bioreactor design described here overcomes these problems by incorporating a gas permeable polydimethylsiloxane (PDMS) shell, which decouples the bulk medium perfusion from the supply of oxygen. This allows a reduced perfusion flow rate or even a single pass medium supply. Fused polystyrene pellets are used to construct a scaffold, that is subsequently air plasma treated to generate charged functional groups on the surface, which promotes cell attachment similar to commercial tissue culture polystyrene (TCP) [15]. Bubble formation within the bioreactor caused by pressure drops and temperature changes across the bioreactor was prevented by pressurizing the waste reservoir to 2 PSI. The system was initially optimised using an immortalized murine MSC population, and then the system was demonstrated to be suitable for the direct isolation of pMSCs from placental tissue digest and subsequent expansion.

## Materials and Methods

### Single Pass Small-Scale Bioreactor Design

**Fig 1:**
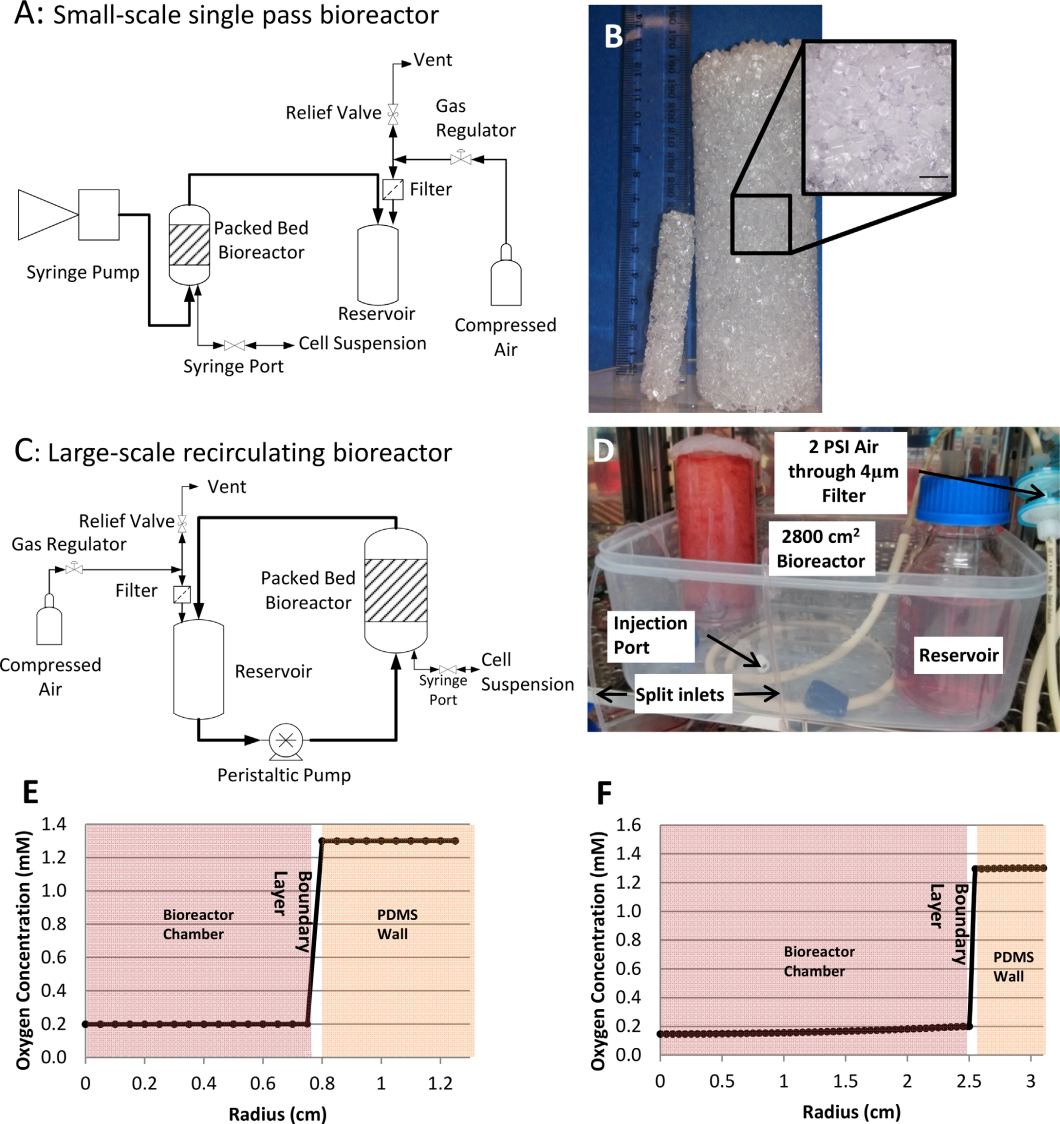
Description of packed bed bioreactor and results from the steady state oxygen mass balance. (A) Process flow diagrams of the single pass small-scale 160cm^2^ bioreactor and (C) recirculating perfusion for the large-scale 2800cm^2^ bioreactor. (B) Picture of 160 cm^2^ left and 2800 cm^2^ right bioreactor scaffolds made from 3 mm polystyrene pellets (scale bar is 10 mm). (D) 2800 cm^2^ bioreactor inside the incubator. (E) Mass transport model result for radial oxygen diffusion with cell density of 100,000 cells/cm^2^ in small-scale (160 cm^2^) and (F) large-scale (2800 cm^2^) packed bed bioreactors.

This system contained a 1.5 cm diameter by 7.5 cm long scaffold providing a total surface area of 160 cm^2^, connected to a single pass circuit (Fig 1A). A 5 mm thick polydimethylsiloxane (PDMS, Dow Corning, MI, USA) tube was moulded to just fit the polystyrene scaffold with an additional 1 cm head space to function as a bubble trap. Perfused medium was driven by a syringe pump (New Era Pump Systems Inc., NE-1800, Farmingdale, NY, USA) that was maintained outside the incubator. Medium was firstly perfused through a 30 cm length of 16 mm diameter silastic tubing (Cole-Parmer, IL, USA) to enable conditioning of the medium before it entered the bioreactor flow cell. The rest of the tubing set was constructed of 1.6 mm Masterflex PharMed BPT tubing (Cole-Parmer, IL, USA).

### Recirculating Large-Scale Bioreactor Design

A recirculating system was implemented as shown in the process flow diagram (Fig 1C). The large-scale bioreactor is a 5 cm diameter by 12 cm scaffold, providing a total surface area of 2800 cm^2^, encased in a 5 mm thick PDMS tube. The peristaltic pump (Watson Marlow Alitea, 403V/VM 4, Stockholm, Sweden) was located outside the incubator. A single 3 mm diameter Masterflex PharMed BPT tubing passed through the peristaltic pump and was expanded into two 30 cm lengths of 1.6 mm diameter silastic tubing to allow conditioning of the medium for the inlet. This was then further expanded into the four 1.6 mm inlet holes into the bioreactor. Medium then left the bioreactor via the four 1.6 mm outlet holes, reducing firstly into two 30 cm lengths of 1.6 mm diameter silastic tubing and finally one 3 mm diameter Masterflex PharMed BPT tubing into the reservoir (Fig 1E).

### Large Surface Area Polystyrene Scaffold

Scaffolds are constructed from 3 mm diameter by 2.5 mm length fused polystyrene cylindrical pellets (a generous gift from Paul Reynolds of Styron, PA, USA). The pellets were fused together by passing Acetone through a mould with a 1.5cm diameter for the small-scale and a 5 cm diameter for the large-scale. The surface area of the scaffold was calculated by using the mass of the column and the volume estimate was confirmed by measuring the void volume.

Details of the plasma reactor used to treat the polystyrene scaffolds have been reported previously [16]. In brief, the plasma reactor was loaded with the untreated scaffold columns, sealed and pumped under vacuum to a base pressure of 7x10^-3^ mbar. Air was then introduced into the reactor at a flow rate of 4 cm^3^/min, while the chamber was rotating at approximately 10 revolutions per minute. Plasma was ignited at 50 W for a total period of 10 minutes.

### Prevention of Bubble Formation in Bioreactor Systems

To prevent bubble formation due to pressure drop and temperature changes in the system, the system was pressurised with air at 2 PSI (13.79 kPa) using a low pressure two stage gas regulator (Gascon Systems, Thornbury, Vic, Australia) and a pressure safety valve set at 2 PSI (Generant, Butler, NJ, USA).

### Air Plasma Treated Polystyrene Scaffold Characterisation

Surface analysis was carried out using Axis Ultra DLD spectrometer (Kratos, Manchester UK.) using a monochromatic Al Kα X-ray source operating at 225 W, which corresponds to an energy of 1486.6 eV. The area of analysis was 0.3 x 0.7 mm and an internal flood gun was employed to minimize the charging of the samples. The survey spectra were collected at a dwell time of 55 ms with 160 eV pass energy at steps of 0.5 eV with three sweeps. The collected data was then analysed and processed using CasaXPS (ver.2.3.16 Casa Software Ltd. ^**®**^) utilizing Shirley baseline correction. To compensate for the charging effects, the C-C peak was offset to 284.8 eV in all spectra. Finally, all atomic percentages were mathematically rounded to one digit after the comma.

### pMSC Isolation and Cell Culture

Placenta was obtained from full term elective Caesarean sections with written patients’ consent and ethics approval from Queensland University of Technology Human Ethics Committee (1000000938). The method used to isolate pMSCs from the placenta has previously been reported in [6,17], it results in a product of <1% CD45^+^ and >90% CD73^+^/CD105^+^. Briefly, 10 g of placental tissue cut into 5 mm pieces were digested in a 50 mL falcon tube with DMEM low glucose (Invitrogen), 100 Units/mL C ollagenase I (Worthington) and 2.5 μg/mL DNase I (Roche) that was incubated at 37°C for 2 hours on a shaker (250 RPM). The cell supernatant was passed through a 70 μm cell strainer (BD Falcon) and was centifuged at 400g for 5 minutes. The supernatant was removed and the cell pellet resuspended in PBS and mononuclear cells were then isolated using a Ficoll-Paque (GE Healthcare) density gradient at 400 *g* for 30 minutes. The mononuclear layer was collected and washed with PBS and finally the cells were resuspended in low glucose DMEM supplemented with 10% fetal bovine serum (Thermofisher) and 100 units/mL penicillin, and 100 μg/mL streptomycin (Life Technologies) (referred to DMEM 10% FBS from here on) and seeded into a single T175 flask. The cells were then incubated in 20% oxygen, 5% CO_2_ at 37°C for 24 hours to allow adherent cells to attach. The flask was washed and fresh medium was added. The flask was placed into a hypoxic incubator (2% oxygen and 5% CO_2_). This process was standardised in our group for MSC isolation procedures [18]. The medium was replaced twice a week until the monolayer was 80–90% confluent before passaging. The pMSCs were harvested and cryogenically stored after passage one.

Primary green fluorescence protein labelled mouse mesenchymal stromal cells (GFP-mMSCs, generous gift from Mater Medical Research Institute) were isolated from the bone marrow of UBI-GFP/BL6 mice in accordance with The University of Queensland Animal Ethics Committee, previously published and described in [19,20]. The GFP-mMSCs were maintained in DMEM 10% FBS and incubated in standard cell culture conditions (37°C and 5% CO_2_).

### Comparison of Air Plasma Treated Polystyrene and Commercial Tissue Culture Plastic on Cell Growth Rate and Attachment

Non-tissue culture treated polystyrene 60 mm Petri dishes (Sigma-Aldrich) were plasma treated in the same manner as the polystyrene scaffolds above. To assess cell attachment, the air plasma treated Petri dishes and T75 flasks (Nunc) were seeded at 2000 cells/cm^2^ with GFP-mMSCs in DMEM 10% FBS. The cells were permitted to attach for one and a half hours. The adhered cells were detached and then counted on an FC 500 flow cytometer (Beckman and Coulter, USA) using flow cytometry counting beads (Beckman and Coulter, USA). To compare the growth rate, plasma treated Petri dishes and T75 flasks were seeded with GFP-MSCs at a density of 1000 cells/cm^2^ and incubated for 3 days in DMEM 10% FBS. The cells were then harvested and counted via flow cytometry as described above.

### Expansion of GFP-mMSCs in the Packed Bed Bioreactor

The 160 cm^2^ bioreactor was seeded at 1000 cells/cm^2^ of GFP-mMSCs suspended in DMEM 10% FBS. To distribute the cells evenly, the bioreactor was placed on a tube rocker roller set at 5 RPM for 10 minutes, followed by 5 minutes of rest in an incubator and repeated for 3 hr. As a 2D control, GFP-mMSCs were seeded at a density of 1000 cells/cm^2^ in T175 flasks (Nunc). The GFP-mMSCs were grown either under static conditions or perfusion (5 mL/day) in the bioreactor. The cell number was quantified every two days by replacing the medium with a 1:50 dilution of AlamarBlue in DMEM 10% FBS medium and incubated for three hours. The reacted AlamarBlue medium was replaced with fresh DMEM 10% FBS and the culture was continued. The fluorescence signal of the AlamarBlue in medium was measured in a plate reader (FLUOstar Omega, BMG Labtech, Germany) along with a cell titration to infer cell numbers using excitation and emission filters of 544 and 590.

### Packed Bed Bioreactor Harvest and Harvest Efficiency

The bioreactor was washed with one reactor volume of PBS and replaced with one reactor volume of 0.01% Trypsin EDTA (Gibco). The Bioreactor was then incubated (5% CO_2_ and 37°C) for 15 minutes. The detached cells were removed by first draining the Trypsin solution and then pumping through three reactor volumes of DMEM 10% FBS. Live cells numbers were determined by an automated cell counter (Bio-Rad TC20, CA, USA) using Trypan Blue. This number was compared to the Alamar Blue result to estimate the percentage of live cells removed from the bioreactor.

### Packed Bed Bioreactor Imaging

For direct imaging of the GFP-mMSCs attached to the 160 cm^2^ column at the end of culture, the column was removed and fluorescence microscopy (Nikon Eclipse Ti-u with a Nikon Digital Sight Ds-Qimc camera, Japan) was used to image GFP expressing cells.

To image the whole bioreactor using the IVIS Imaging System 200 series (Caliper, PerkinElmer, MA, USA), the cells were fixed with 4% paraformaldehyde and were stained with 10 μg/mL propidium iodide (PI, Invitrogen) for 10 minutes. The bioreactor was then washed with PBS and stored on ice prior to imaging. As negative controls, bioreactors containing no cells underwent the same fixing and staining process. The bioreactors were imaged with an excitation filter of 520 nm and an emission filter of 620 nm. The thresholds were adjusted to remove background auto florescence.

### Pre-Isolated pMSCs Expansion in the Packed Bed Bioreactor

The 160 cm^2^ scaffold bioreactor was seeded through the injection port with 1000 cells/cm^2^ of passage four pMSCs suspended in DMEM 10% FBS, using the same seeding process, culture conditions and 2D controls as the expansion of the GFP-mMSCs. However, in the perfusion experiment the cell numbers were quantified by AlamarBlue at the end of the culture. The cells were then harvested and characterised by mesodermal tri-lineage differentiation capacity.

### pMSCs Isolation and Expansion in the Packed Bed Bioreactor

The placental tissue was digested by the same method outlined in the pMSC isolation and cell culture section above. Instead of a density gradient the filtered cell suspension from the 10 g of starting tissue was seeded into the bioreactor or into a T175 flask, using the same seeding method outlined in the GFP-mMSC expansion. After 24 hours, the cell suspension was removed, the medium was replaced and the flow rate was adjusted in the bioreactor to 5 mL/day. Once the pMSC colonies were observed in the T175 flasks, the cells were harvested in both the bioreactors and flasks and reseeded into the same bioreactor or flask to continue the expansion process. The cell number was monitored using the AlamarBlue. The cells where harvested once reaching confluence and characterised by mesodermal tri-lineage differentiation capacity.

### Scale-up of GFP-mMSC in Bioreactor

The 2800 cm^2^ bioreactor was seeded at 1000 cells/cm^2^ with GFP-mMSCs suspended in DMEM 10% FBS using the injection port. The T175 flasks function as a 2D control. The same seeding procedure as per the small-scale expansion experiments was followed. The reservoir was filled with 250 mL of DMEM 10% FBS to give a total medium volume of 360 mL in the circuit (bioreactor volume 110 mL). The medium was pumped in the recirculating circuit at 0.5 mL/min. A 1 mL medium sample was taken from the injection port each day. In addition, at the end of the culture period an additional 1mL sample was taken from the bulk medium in the reservoir. Glucose and lactate concentrations were quantified by the Mater Hospital Pathology laboratory. At the end of expansion, the cell numbers were quantified by the AlamarBlue method.

### Mesodermal Differentiation

Osteogenic differentiation was achieved by culturing pMSCs in induction medium containing high glucose DMEM (HG DMEM, Invitrogen) supplemented with 10% FBS, 10 mM β-glycerol phosphate (Sigma-Aldrich), 100 nM Dexamethasone (Sigma-Aldrich) and 50 μM L-ascorbic acid 2-phosphate (Sigma-Aldrich). The calcium deposits were visualised by staining with AlizarinRed S (Sigma-Aldrich). Alkaline phosphatase (ALP) activity was quantified with p-nitrophenyl Phosphate (Sigma-Aldrich) following manufacturers’ instructions, measuring the absorbance at 405 nm after a 30 minute incubation.

Chondrogenesis was induced using cultures of 200,000 cell pellets grown in HG DMEM supplemented with 110 μg/mL sodium pyruvate (Invitrogen), 10 ng/mL recombinant human Transforming Growth Factor β1 (TGF-β1, Peprotech), 100 nM dexamethasone, 200 μM ascorbic acid 2-phosphate, 40 μg/mL L-proline (Sigma-Aldrich) and 1% ITS-X (Invitrogen). 75% of the chondrogenic medium was changed every two days, the collected medium was stored at -20°C for subsequent glycosaminglycan (GAG) analysis. GAG was visualized by staining frozen sections of the cell pellets with Alcian Blue (Sigma-Aldrich). GAG was then quantified by first digesting the pellets in papain and then staining with 1,9-Dimethyl-methylene blue zinc chloride double salt (Sigma-Alrich).

Adipogenic differentiation was induced over 2 weeks with HG DMEM supplemented with 10% FBS, 10 μM insulin (Invitrogen), 1 μM dexamethasone, 200 μM indomethacin (Sigma-Aldrich) and 500 μM 3-isobutyl-1-methyl-xanthine (Sigma-Aldrich). Lipid droplets were visualised by staining with Oil Red O (Sigma-Alrich) and were quantified with an adipogenensis detection kit (Abcam) following the manufacturer’s protocol.

The DNA was quantified by PicoGreen dsDNA Reagent Kit (Invitrogen) following the manufacturer’s protocol.

### Steady-state Oxygen Mass balance

A mass balance calculation crudely estimates the concentration of oxygen at any point along the radius by a simple source sink method. A more sophisticated theoretical treatment was applied, but will be published elsewhere. It was assumed that there was no axial flow and that oxygen only diffused radially; no axial diffusion was considered. Eq 1 describes the concentration of oxygen in the bioreactor vessel, while Eq 2 describes the oxygen concentration in the wall of the bioreactor. C_R1_ (mM) is the concentration at the inner bioreactor radius (R_1_,cm) which is the solubility of oxygen in the medium (0.2 mM), C_R2_ is the concentration at the outer bioreactor R_2_ radius which is the solubility of oxygen in PDMS (1.3 mM) [21], C_r_ is the concentration at any point of the radius r, K is the specific volumetric uptake of oxygen (mM/cm^3^/s), derived from bioreactor dimensions and MSCs oxygen uptake rate of 2.5x10^-17^ mM/s/cell [22], D_R_ is the corrected diffusion coefficient of oxygen within the bioreactor vessel and D_pdms_ is the diffusion coefficient of PDMS (5.0x10^-5^ cm^2^/s [21]).

$$C_{r}=C_{R_{1}}-\frac{K}{4D_{R}}(R^{2}-r^{2})-\frac{K{R_{1}}^{2}}{2D_{pdms}}\ln\;\left(\frac{R_{2}}{R_{1}}\right)\;0\leq r\leq R_{1}$$ (1)

$$C_{r}=C_{R_{2}}-\frac{K{R_{1}}^{2}}{2D_{pdms}}\ln\;\left(\frac{R_{2}}{r}\right)\;R_{1}\leq r\leq R_{2}$$ (2)

However, as the bioreactor vessel space is filled with non-gas permeable particles, a correction factor (Eq 3) was applied to the diffusion coefficient based on the voidage (ε), sphericity (φ, Eq 5) and tortuosity (τ, Eq 4). The voidage, calculated as the difference between the empty volume of the bioreactor and the liquid volume injected into the bioreactor, was found to be 0.4.

$$D_{R}=\frac{\varepsilon }{\tau }D_{m}$$ (3)

Correction factor for oxygen diffusion coefficient in medium Dm (3.290x10^-05^ cm^2^/s [23]), is a function of voidage (ε) and tortuosity (τ).

$$\tau =\frac{1.23{\left(1-\varepsilon \right)}^{\frac{4}{3}}}{\varepsilon \times \varphi^{2}}$$ (4)

Tortuosity is a function of the voidage and sphericity (***φ***). Equation taken from [24].

$$\varphi =\frac{(36\times \pi \times n{\left(1-\varepsilon \right)}^{2}{)}^{\frac{1}{3}}}{a}$$ (5)

Sphericity is calculated based on the number of particles (n), the voidage (ε) and the surface area to volume ratio (a). Equation taken from [24].

To calculate the shear stress from the medium perfusion, the pressure drop (ΔP) was first calculated by the Ergun equation:

$$\Delta P=\frac{150\mu {\left(1-\epsilon \right)}^{2}V_{S}L}{\varepsilon^{3}D_{p}^{2}}+\frac{1.75(1-\varepsilon )\rho V_{s}^{2}L}{\epsilon^{3}D_{p}}$$ (6)

The viscosity (μ) of the medium is 0.008 poise [25], the density of the medium (ρ) is 0.99 g/cm^3^[26], V_s_ is the superficial velocity, D_p_ is the diameter of the particles and L is the length/height of the packed bed. To calculate the shear force (τ) the pressure drop was multiplied by the cross sectional area of the fluid flow ($\frac{\pi D^{2}\varepsilon }{4}$) and divided by the surface area of the fluid contacting the scaffold (growth area of the scaffold, i.e. 160 cm^2^ and 2800 cm^2^).

### Statistics

Results are expressed as means and standard deviations of four biological replicates. Differences were determined by T-test using SPSS statistics (ver 17, SPSS Inc, Chicago) and values of p≤0.01 were considered significant.

## Results

### Steady-state Oxygen Mass balance

The bioreactor mass transport model was used to estimate the steady-state oxygen concentration along the radius at a cell concentration five times the maximum confluence of MSCs (100,000 cells/cm^2^). The mass balance predicted no significant oxygen gradient in the small-scale 160 cm^2^ bioreactor (Fig 1E). However, in the larger 2800 cm^2^ bioreactor, a drop in oxygen concentration of 0.2 mM to 0.146 mM in the centre was predicted (Fig 1F). As the PDMS wall offered negligible resistance to oxygen diffusion in both configurations, the only factor limiting the oxygen concentration in the bioreactor was the oxygen solubility in the medium.

The Ergun equation was used to calculate the shear due to fluid flow in the bioreactor. The hydrodynamic shear from the medium perfusion in the small bioreactor (5 mL/day) and large bioreactor (0.5 mL/min) was calculated to be 9.5x10^-5^ Pa and 1.25x10^-3^ Pa, respectively.

### Plasma Treated Polystyrene Scaffold Characterisation

**Fig 2:**
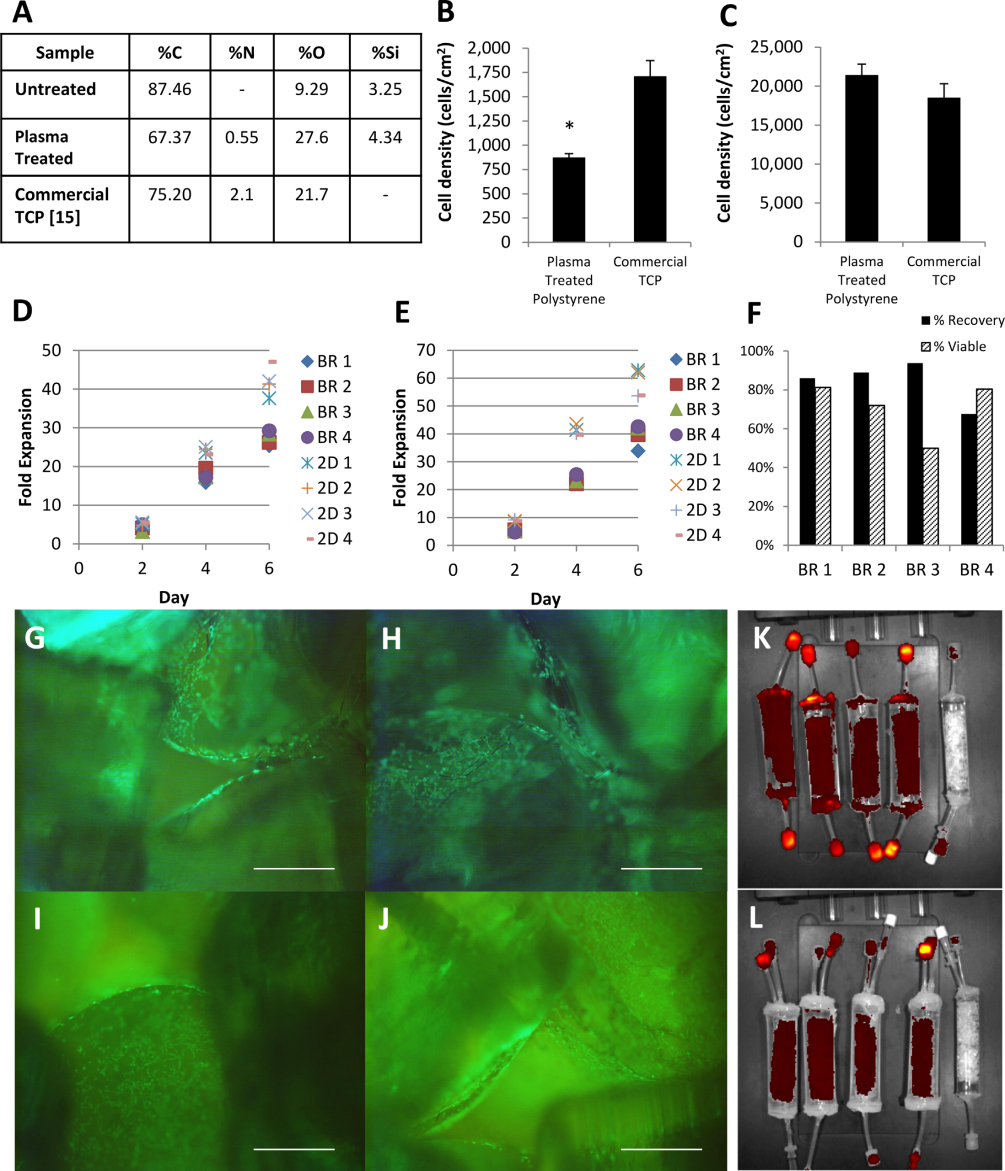
Characterisation of plasma modified surface and GFP-mMSCs expansion in our packed bed bioreactor in static and perfusion conditions. (A) Surface composition of the air plasma treated polystyrene scaffold determined by XPS. (B) GFP-mMSC attachment after one and half hours, initially seeded at 3000 cells/cm^2^ (n = 4). (C) The growth after 3 days of culture of GFP-mMSC seeded at 1000 cells/cm^2^ (n = 6). (D) Growth of GFP-mMSC in the bioreactor (BR) under static conditions (n = 4) and (E) under 5 mL/day perfusion (n = 4). (F) Cell harvest recovery and viablity from the bioreactor. (G, H, I and J) Fluorescent microscopy showed that the GFP-mMSC attached to the scaffold (scale bar is 500 μm). (K) IVIS imaging of the fluorescent intensity of PI stained GFP-mMSC in the bioreactor under static and (L) 5 mL/day perfusion conditions. IVIS images are a red (low) / yellow (high) heat map of fluorescent intensity.

The rotary air plasma treatment greatly increased the charged oxygen groups on the surface on the polystyrene (Fig 2A), providing a similar growth surface chemical composition to commercial TCP [15]. However, high amounts of silicon and oxygen contamination in the untreated polystyrene was detected. This was likely because the PDMS mould used to make the scaffold is known to leach unreacted polymer which contains silicon and oxygen [27].

The attachment and growth of GFP-mMSC were compared on our plasma treated surface in 2D and commercial TCP. Only half of the 3000 cells/cm2 seeded attached to the plasma treated surface after one and half hours (Fig 2B). However after 3 days the cell number on the plasma treated surface was equivalent to commercial TCP (Fig 2C). Following this observation, the seeding period for the bioreactor experiments was extended to 3 hours to allow more robust cell attachment.

### GFP-mMSCs Expansion in Small-scale Packed Bed Bioreactor

The GFP-mMSCs growth rate in the bioreactor was significantly less than the 2D controls under both static and perfusion conditions (Fig 2D & 2E) with the doubling time of 30.19±0.6 hr and 26.48±0.49 hr, respectively for static conditions and 27.18±0.8 hr and 24.59±0.53 hr, respectively under perfusion conditions (5 mL/day). The cells were harvest from the bioreactor with an efficiency of 84%±11% with a cell viability of 71%±15% (Fig 2F).

The cells formed a monolayer on the fused pellets and maintained a spindle-like morphology. In addition, the cells were observed to grow across the connection between the beads (Fig 2G, 2H, 2I & 2J). IVIS imaging used the fluorescent intensity of the PI stained cells to estimate the global distribution of cells within the bioreactor. Under both static and perfusion conditions, fluorescence intensity was homogeneous within the scaffold (Fig 2K & 2L), suggesting that the cells could be homogeneously distributed in the scaffold.

### Human Placental MSCs Expansion in Small-Scale Packed Bed Bioreactor

**Fig 3:**
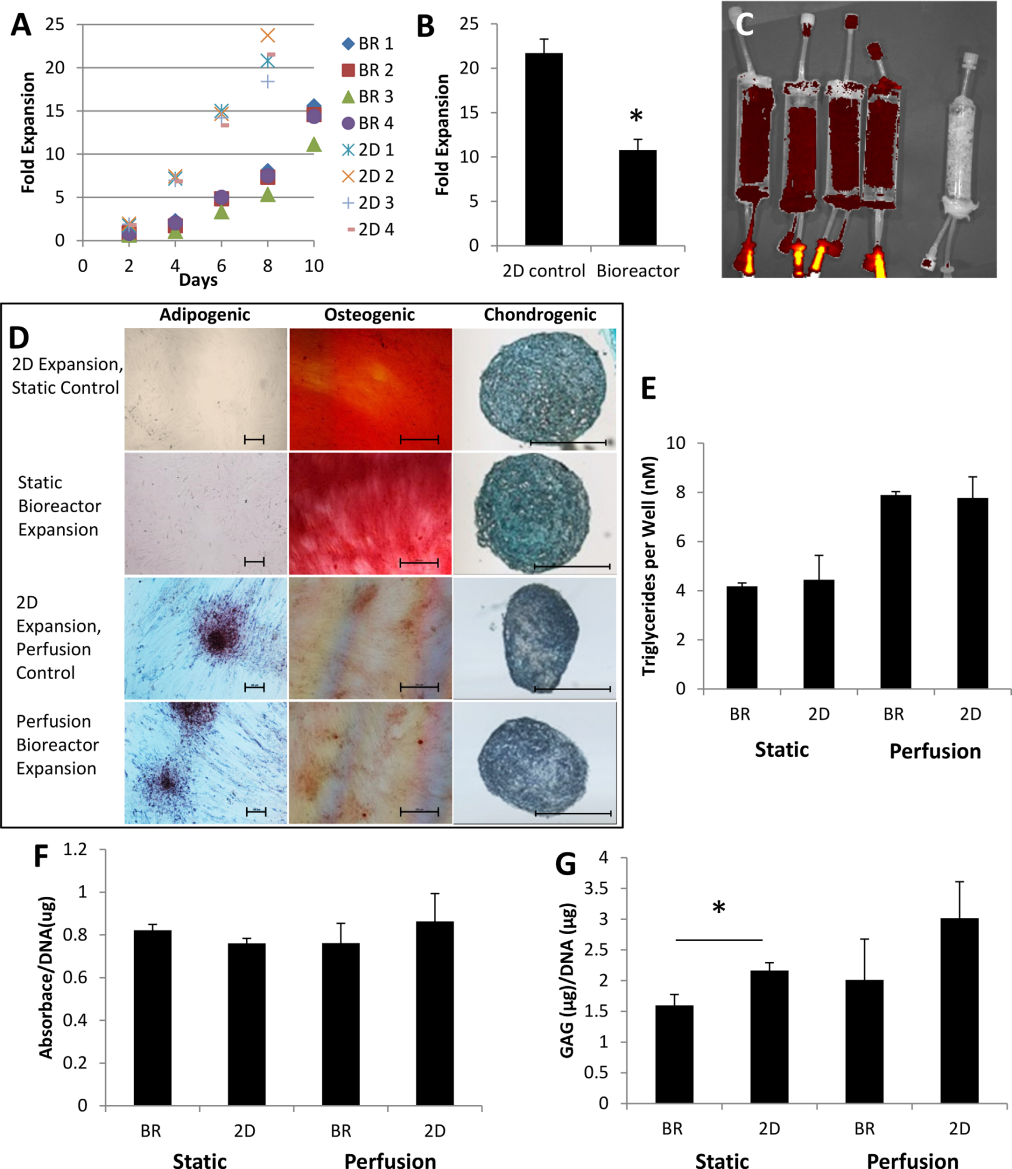
Pre-isolated passage four pMSC expanded in our bioreactor in static and perfusion conditions. (A) pMSC expansion in the small-scale 160 cm^2^ bioreactor (BR) in static (n = 4) and (B) 5 mL/day perfusion (n = 4), with the 2D controls (2D). (C) IVIS imaging of PI stained pMSC under perfusion conditions. IVIS images are a red (low) / yellow (high) heat map of fluorescent intensity. (D) Two week tri-lineage mesodermal differentiation induction of bioreactor expanded pMSC and 2D controls down the adipogenic (Oil Red O, 10x, scale bar is 100 μm), osteogenic (Alizarin Red, 5x, scale bar 500 μm) and chondrogenic (Alcian Blue, 10x, scale bar 500 μm) lineages. Quantification of (E) triglycerides (n = 4), (F) ALP activity (n = 4) and (G) GAG production.

Passage 4 human pMSCs were expanded under static conditions and perfusion conditions. The doubling time of bioreactor expanded cells took longer than the 2D controls (T175 flask), 63.5±1.5 hr and 43.7±1.5 hr, respectively for static (Fig 3A) and 49.2 ± 2.6 hr and 37.9 ± 0.9 hr, respectively for perfusion (Fig 3B). A single end point quantification protocol after seven days of expansion was adopted in the perfusion experiments, due to the disruption caused to the cells by taking a measurement every two days. This resulted in a reduced difference in the doubling times between perfused bioreactor grown cells and the 2D controls. IVIS images of pMSCs expanded under perfusion conditions displayed an even fluorescent intensity from the scaffold; a suggestion of homogeneous distribution of cells (Fig 3C). Note that due to the residual medium trapped in the syringe port resulted in a high intensity fluorescent signal detected by the IVIS.

The cells were characterised by mesodermal tri-lineage differentiation There was no difference between the bioreactor expanded cells in both static and perfusion conditions and the corresponding 2D controls when stained with Oil Red O, AlizarinRed S or Alcian Blue (Fig 3D). This was confirmed with no difference in the triglycerides amount and ALP activity (Fig 3E & 3F). However, there was significantly less GAG production in the cells expanded in the bioreactor under static conditions relative to the 2D control (Fig 3G).

### Isolation and Expansion of Placental MSC Directly in Small-Scale Packed Bed Bioreactor

**Fig 4:**
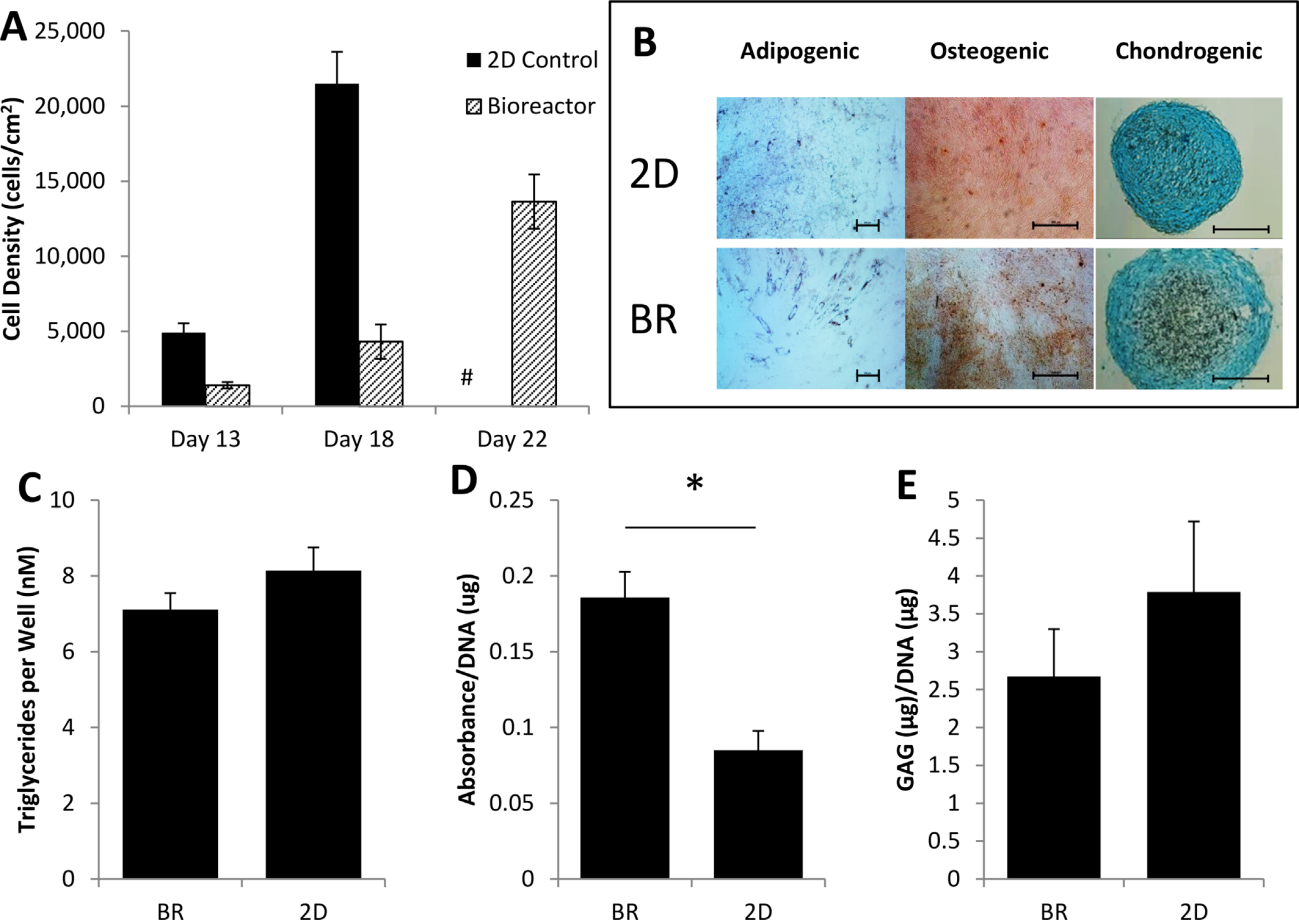
Isolation of pMSCs from digested placental tissue in our packed bed bioreactor. (A) cell growth of pMSCs isolated directly from a placenta in the 160 cm^2^ packed bed bioreactor undergoing 5 mL/day perfusion (n = 4). On day 13, the cells were redistributed back into the same flask or bioreactor. # indicating no 2D control result was measured as the flask was harvested on day 18. (B) Two week mesodermal tri-lineage differentiation induction of bioreactor expanded pMSC and 2D controls down the adipogenic (Oil Red O, 10x, scale bar is 100 μm), osteogenic (Alizarin Red, 5x, scale bar 500 μm) and chondrogenic (Alcian Blue, 10x, scale bar 500 μm) lineages. Quantification of triglycerides (n = 4) (C) and ALP activity (n = 4) (D) GAG (E) production compared to its matching 2D control (n = 4).

pMSCs were isolated from 10 g of digested placenta directly into the bioreactor was expanded under 5 mL/day perfusion. The growth rate of pMSCs was slower in the bioreactor compared to the 2D controls (Fig 4A). The pMSC colonies were becoming confluent following 13 days of culture in the 2D controls, at this point the cells were re-seeded back into the same flask or bioreactor, and the expansion was continued. At day 18 the 2D controls reached confluence and were harvested and characterised by mesodermal tri-lineage differentiation. The bioreactor pMSCs’ cultures were harvested at day 22.

The qualitative differential potential of the osteogenic, chondrogenic and adipogenic lineages were similar as shown by staining between the bioreactor expanded pMSCs and the 2D controls (Fig 4B). However, when quantified (Fig 4C, 4D & 4E) there was a significant difference in ALP activity between bioreactor isolated and expanded cells compared to the 2D controls.

### Scale-up of the Packed Bed Bioreactor

**Fig 5:**
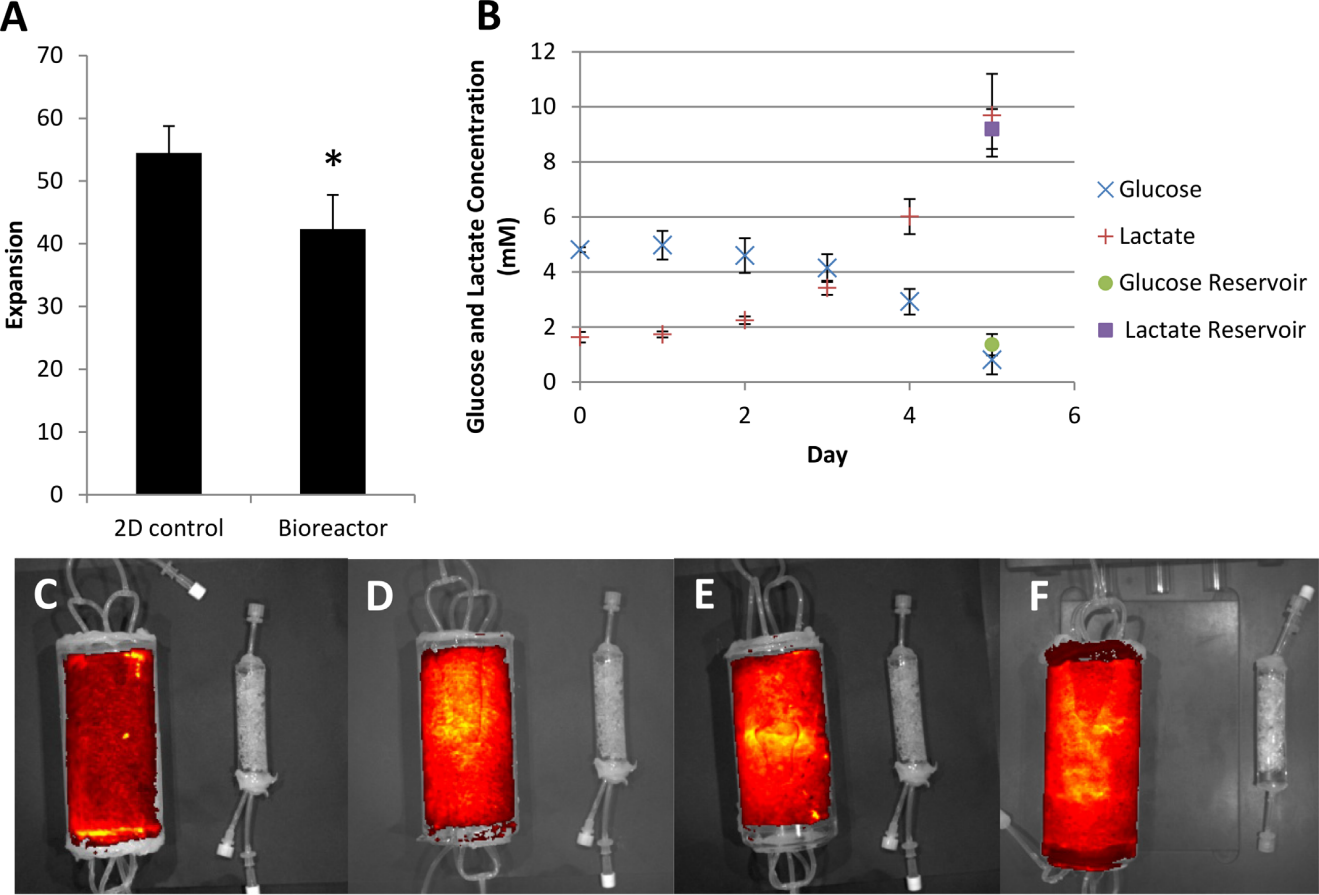
GFP-mMSC expansion in a scaled-up packed bed bioreactor (A) The fold expansion of GFP-mMSC in a scaled-up bioreactor under 0.5 ml/min perfusion with T175 flask control (n = 4). (B) Glucose and lactate levels in the bioreactor (n = 3). (C, D, E & F) IVIS imaging of the fluorescent intensity of PI stained GFP-mMSC in the bioreactor. IVIS images are a red (low) / yellow (high) heat map of fluorescent intensity.

GFP-mMSC was used to demonstrate the scale up potential of a larger 250 mL vessel that provided 2800 cm^2^ of growth surface. The cell growth was significantly slower in the bioreactor than in the 2D controls, with doubling times of 21.5 ± 0.1 hr and 20.8 ± 0.4 hr, respectively (Fig 5A). The medium perfusion rate was increased to 0.5 mL/min from the small-scale experiments to provide adequate glucose to the cells. The glucose levels were nearly depleted in the bioreactor and the reservoir by day 5 (Fig 5B), suggesting either that the medium must be changed every three to four days or that a larger medium reservoir is required.

The IVIS imaging showed heterogeneous fluorescence intensity, implying that the seeding method is not as robust when scaled-up to the large-scale bioreactor compared to the small-scale bioreactor (Fig 5C, 5D, 5E & 5F).

## Discussion

The packed or fixed bed bioreactors, like many other bioreactor designs, rely either on the perfusion or mixing of the medium to provide the two limiting metabolites, glucose and oxygen. Due to the low oxygen solubility, the perfusion or mixing rates must be high to meet the oxygen demand from the expanding cells and to prevent depletion and significant gradients [14,28]. Such high perfusion rates can potentially cause shear stress on the cells, which has been shown to reduce cell growth rate and induce differentiation. A shear force of 0.015 Pa has been reported to up-regulate osteogenic pathways in human bmMSCs [12–14,29]. Our bioreactor employed a gas permeable wall to decouple the oxygen supply from the bulk medium perfusion, resulting in lower flow rates and putatively decreasing the shear force to which the cells are exposed to an estimated 9.5x10^-5^ Pa for the small-scale bioreactor and 1.25x10^-3^ Pa for the large-scale bioreactor. In addition, the lower medium volume required for tissue culture would represent a significant cost saving.

The overall growth rate of pMSCs expanded in the bioreactor was less than that observed for traditional flask expanded cells (Figs 2E & 3B), and was similar to bmMSCs and pMSCs expansion growth rates reported for other bioreactor designs [6,8,30,31]. Medium perfusion enhanced the growth rate of both GFP-mMSC and pMSC, consistent with literature observations [6,8,30,31].

This impaired bioreactor growth rate relative to traditional tissue culture flasks could be attributed to surface chemistry and geometry of the scaffold or to the method used to seed the bioreactor. However, our air plasma treated polystyrene surface provided a comparable surface to commercial grade tissue culture plastic (Fig 2A), and the growth rate was comparable to commercial TCP (Fig 3C). Thus, the impaired growth rate observed in the bioreactor cannot be entirely attributed to chemical differences in surface composition. The inefficiencies in the cell seeding process can also strongly affect the growth rate. A noticeable uneven cell distribution was observed in the scaled-up bioreactor (Fig 5C, 5D, 5E & 5F). The geometric features of the bioreactor scaffold surface alter the contact inhibition characteristics of cell colonies, which would be likely to reduce the observed growth rate. In addition, the collision rate during seeding, defined as the rate at which cells contact the scaffold, would be greatly reduced on these 3D structures in dilute cell suspension [11]. Of the many bioreactor seeding methods reported in the literature, no method was completely efficient [14,32–34].

Despite the significant decrease in growth rate in our bioreactor, the pMSCs maintained the capacity to differentiate down the osteogenic, adipogenic and chodrogenic lineages. No differences in mesodermal tri-lineage differentiation were observed under the perfusion expansion protocol (Fig 3E, 3F & 3G). However, the GAG production in static bioreactor-expanded cells diminished relative to 2D controls. As preconditioning MSCs to a higher oxygen concentration reduces their capacity to differentiate down the condrogenic linage, reduced GAG production could be explained by a higher oxygen concentration present in the bioreactor compared to the traditional tissue culture flask, [35,36]. The liquid layer in a traditional tissue culture flask offers greater resistance to oxygen diffusion resulting in a lower concentration of oxygen at the cell layer [37]. MSCs in the bioreactor located at the liquid-wall interface are exposed to far higher oxygen concentration.

To further characterise the bioreactor’s effect on MSCs stemness, and to demonstrate the flexibility of the design, pMSCs were isolated from a digested placenta directly into the bioreactor. The pMSCs produced from this protocol maintained their potential to differentiate down the adipogenic, osteogenic and chondrogenic lineages (Fig 4B). However, the bioreactor-expanded cells exhibited greater ALP activity than the analogous traditional tissue culture flask expanded pMSCs (Fig 4D). During isolation, large amounts of red blood cells and other dead cell debris could not be removed and remained within the bioreactor column. As dead cells have been reported to contribute to, and possibly even induce, osteogenesis in bmMSCs [38], such debris could pre-condition bioreactor-isolated pMSCs toward osteogenic differentiation. The manual syringe driven process used in this experiment made removing the cell debris from the bioreactor difficult. A steady flow rate was difficult to achieve using this manual method, so the flow rate was kept low to avoid detaching the cells from the scaffold. Automated medium flow in future generations of the bioreactor will help prevent this build-up of cell debris, as a controlled continuous flow will allow higher flow rates without detaching the cells from the scaffold. An increased flow rate used in the expansion process in the scaled-up bioreactor will also actively remove cell debris.

Although the growth rate of pMSCs in the bioreactor was less than the cells grown on tissue culture plastic, the few extra days required for MSCs to reach confluence in the packed bed bioreactor represents an acceptable trade-off to have the potential for an automatable method to digest, isolate and expand pMSCs in a single closed bioreactor system. An isolation/expansion process based on our design, although performed manually here, could be easily automated [6]. Thus, a single automated bioreactor imbedded with an automated isolation protocol might be developed to digest, isolate and expand pMSCs for clinical use with single procedure in a completely closed system. Additionally, the theoretical scaffold height in our bioreactor design is now defined by the much higher glucose concentration, therefore the potential radius of our bioreactor is only limited by the radial diffusion of oxygen [8,14]. The radius limitation might be overcome by embedding coils within the packed bed scaffold, similar to those found in isothermal chemical reactors, constructed of gas permeable PDMS or silicon to optimise oxygen mass transfer. Although our scale-up described here focused only on increasing the actual reactor size, decreasing the pellet size to 0.5 mm and the total surface area to 1.8 m^2^ would be far more efficient in increasing the reactor capacity. This potentially could support 2 x 10^8^ cells using the same dimensions as our larger bioreactor (5 cm diameter and 12 cm height).

## Conclusion

The packed bed bioreactor designed to decouple the oxygen transport from the bulk nutrient supply from the medium flow operates efficiently at a lower perfusion rate, resulting in lower shear stress acting on the cells. We observed a 10 fold expansion of pMSCs in the bioreactor after a one week culture, while still maintaining their differential potential. In addition, pMSC isolated and expanded directly onto the bioreactor still maintained their mesodermal tri-lineage differentiation potential. This design is scalable and potentially automatable. Although the growth rate of pMSCs in the bioreactor was less than the cells grown on tissue culture plastic, the few extra days required for MSCs to reach confluence in the packed bed bioreactor represents an acceptable trade-off to have the potential for an automatable method to digest, isolate and expand pMSCs in a single closed bioreactor system.
